# Case report: Focal heterotopic ossification in paravertebral muscles as a cause of neurogenic lameness in a dog

**DOI:** 10.3389/fvets.2024.1335175

**Published:** 2024-05-23

**Authors:** Ivo Hajek, Marco Rosati, Kaspar Matiasek, Michal Babinsky, Abby Caine, Viktor Palus

**Affiliations:** ^1^Small Animal Referral Centre Sibra, Bratislava, Slovakia; ^2^Section of Clinical & Comparative Pathology, Centre for Clinical Veterinary Medicine, LMU Munich, Munich, Germany; ^3^Veterinary Ambulance, UNI-VET, Levice, Slovakia; ^4^Dick White Referrals, Cambridgeshire, United Kingdom; ^5^Neurovet, Trenčín, Slovakia; ^6^Faculty of Veterinary Medicine, University of Veterinary Sciences Brno, Brno, Czechia

**Keywords:** dogs, mononeuropathies, MRI, heterotopic, ossification, canine

## Abstract

This case report describes a 17-month-old Pudelpointer with recurring motor impairment localized to the left thoracic limb. A neurological exam highlighted lameness in that limb, accompanied by pre-scapular swelling. Radiographs and magnetic resonance imaging detected an osseous structure in soft tissues close to the fifth cervical vertebra, and subsequent surgery uncovered adjacent cervical spinal nerve impingement. Histology of the bony structure revealed heterotopic ossification in paravertebral muscles. Mild bone re-formation at the operating site was detected after a 2-year period, but the patient was asymptomatic. This article reports the first case of heterotopic ossification with spinal nerve entrapment in a dog and adds a new differential diagnosis to the causes of neurogenic lameness in dogs.

## Introduction

Heterotopic ossification (HO), also known as paraosteoarthropathy, myositis ossificans, and heterotopic calcification, is defined as the abnormal formation of mature lamellar extraskeletal bone in soft tissues, such as skeletal muscles, articular capsules, ligaments, and tendons (1–3). In human medicine, HO is divided into two major types—acquired and genetic (4). The acquired form is the most frequent, is closely associated with tissue trauma in 75% of cases, and can be seen after joint surgery, musculoskeletal or central nervous system injury, and burns (5, 6). Many cases have a benign course; however, they may cause inflammation, pain, or functional changes (7). In dogs and cats, the incidence of HO is low, and case reports from the last 50 years are sparse. In dogs, HO was associated with changes around the hip joints or with traumatic events; in cats, the multifocal progressive fibrodysplastic changes resembling human cases have been described (8–21). The aim of this report is to describe the diagnostic imaging and histopathologic features of a canine case of HO lesion in paravertebral muscles with associated spinal nerve impairment.

## Case description

**Figure 1:**
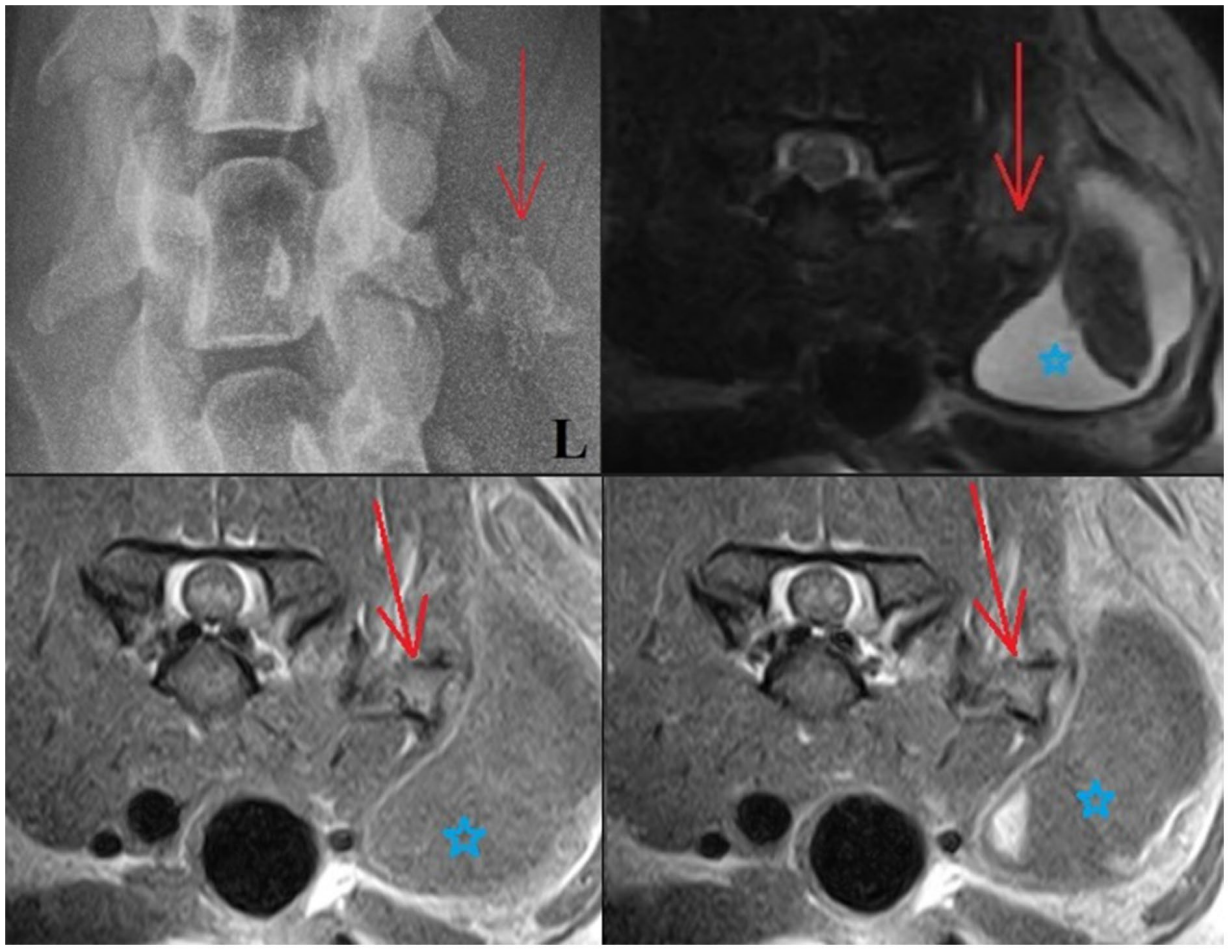
Upper left: Ventrodorsal radiograph of the cervical region: The mineralized lesion is close to the left transverse process of C5 (red arrow), with the blunted tip of the transverse process appearing to mold around the mineralized lesion. Upper right and lower images: T2W, T1W (lower left), and T1W + C (lower right) transverse MRI images: An angular structure is seen in the soft tissues lateral to the left side of the C5 vertebra. This has a signal void thin margin with the appearance of bone cortex and a core that is isointense to bone marrow (red arrow). The surrounding soft tissues are heterogeneous. Note also how there is a large area of surrounding soft tissue swelling cranial to the left shoulder (blue star).

A 17-month-old 25 kg intact female Pudelpointer was referred with intermittent (several months duration) left thoracic limb lameness with no obvious signs of pain. The patient was a pet; there was no history of trauma, and there were no similar symptoms in parents or littermates. Prior episodes of left shoulder edema and abscessation were described, requiring repeated draining, flushing, and antimicrobial therapy. Physical examination during presentation detected left-sided pre-scapular swelling sensitive to palpation. A complete neurologic examination revealed left thoracic limb monoparesis with lameness grade 2/5, decreased flexor reflex, and postural reactions in the affected limb, indicating a lower motor neuron lesion. Neuroanatomic localization was consistent with a left-sided brachial plexus or ventral horn gray matter lesion localized between the sixth cervical (C6) and the second thoracic (T2) spinal cord segments. Differential diagnoses included inflammatory, traumatic, anomalous, neoplastic, and degenerative diseases with local soft tissue reactions. All parameters from the complete blood count (ProCyte Dx Hematology analyzer) and serum biochemical analysis (Cobas c 111 analyzer) were within normal limits. Diagnostic imaging included conventional radiography and MRI of the C1-T2 spine and paravertebral tissues on the left. On radiographs, an irregularly marginated, angular mineral opacity was located adjacent to the left transverse process of the fifth cervical vertebra (Figure 1). This transverse process was blunted and irregular, tessellating with the region of soft tissue mineralization. Marked soft tissue swelling was identified around the mineralization.

An MRI was performed under general anesthesia using a 0.18 T Esaote Vet-MR unit. Multiplanar T2-weighted (T2W), T1-weighted (T1W), and Gradient echo (GE) sequences of the cervicothoracic region were acquired (T1W: TR: 1120, TE: 26, FA: 90, ST: 4; T2W: TR: 3990, TE: 100, FA: 90, ST 4; T2*: TR: 1220, TE: 28, FA: 40, ST: 4). Additional T1W images were performed following intravenous administration of a gadolinium-based contrast medium (Omniscan, GE Healthcare AS, NO, 0.1 mmol/kg). MRI images revealed an angular structure (10 × 11 × 16 mm) seen in the soft tissues lateral to the left side of the C5 vertebra. This had a signal void thin margin with the appearance of bone cortex and a core that was isointense to bone marrow on T2W, T1W, and GE T2* images. This mineralized structure mimicked the appearance of bone yet lay within the muscle belly. The surrounding muscle itself was heterogenous, and there was a large accumulation of fluid content noted in the overlying subcutaneous tissues measuring up to 10 × 2.7 cm—mostly the content of this cystic lesion was T1W hypointense and T2W hyperintense; however, the core was T2W/T2* hypointense (T2W images were partially disrupted by metal artifact caused by the microchip). Some contrast enhancement was noted in the muscle surrounding the mineralized structure, and further enhancement was noted in the right (dependent) aspect of the fluid pocket (Figure 1). Due to the appearance of “bone,” the mineralized structure was considered consistent with HO (with differentials for a mineralized focus including dystrophic mineralization from a trauma, osteochondromatosis, chondroma/sarcoma, paraosteal osteosarcoma, or calcinosis circumscripta). The cystic lesion was considered to be a complex cyst/seroma with a hemorrhagic component, a hematoma, or potentially an abscess.

**Figure 2:**
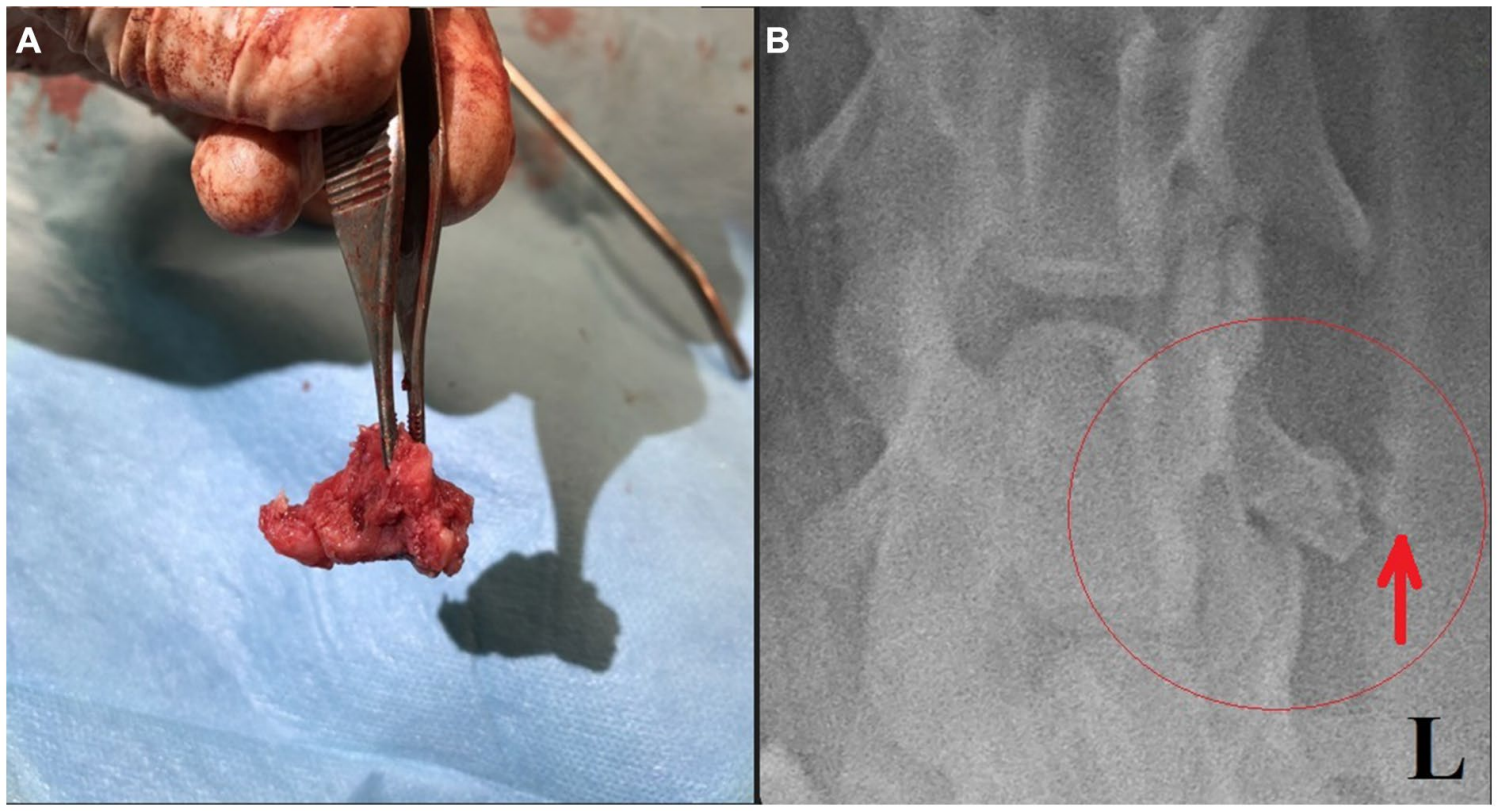
**(A)** Removed bony structure. **(B)** Post-surgical ventrodorsal radiograph of the cervical region with a small amount of residual tissue suspected incomplete lesion capsule removal (red ellipse and arrow).

**Figure 3:**
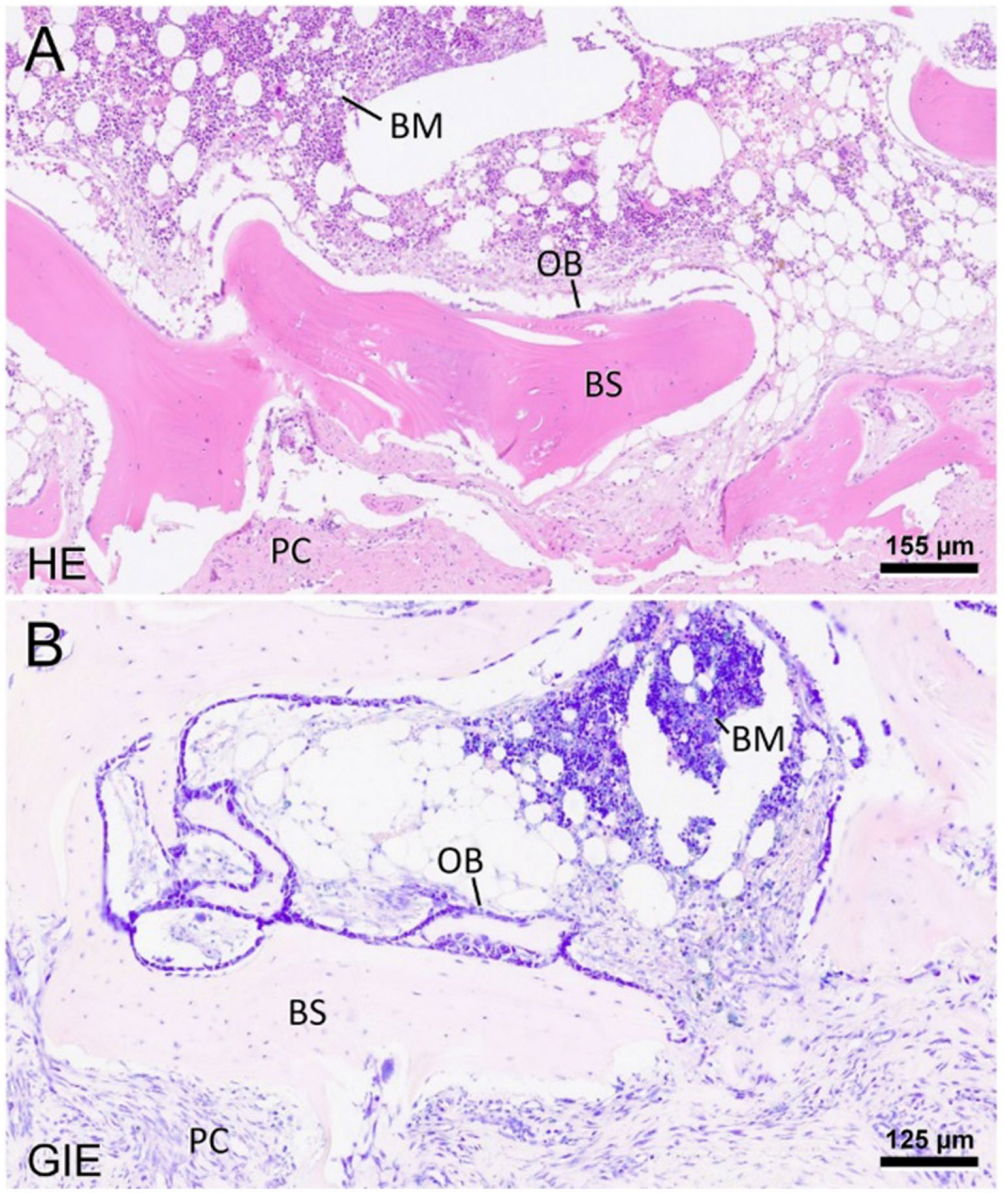
Representative photomicrographs of the heterotopic ossification stained with hematoxylin–eosin **(A)** and Giemsa **(B)**. The mass presents well-differentiated bone spicules (BS) and is confined by a periosteum-like pseudocapsule (PC). Toward the endosteal compartment, there is a layer of osteoblasts (OB). The newly formed bone further contains hematopoietically active bone marrow (BM), interrupted by fat vacuoles. The scale bars indicate the level of magnification. HE: hematoxylin–eosin; Gie: Giemsa.

**Figure 4:**
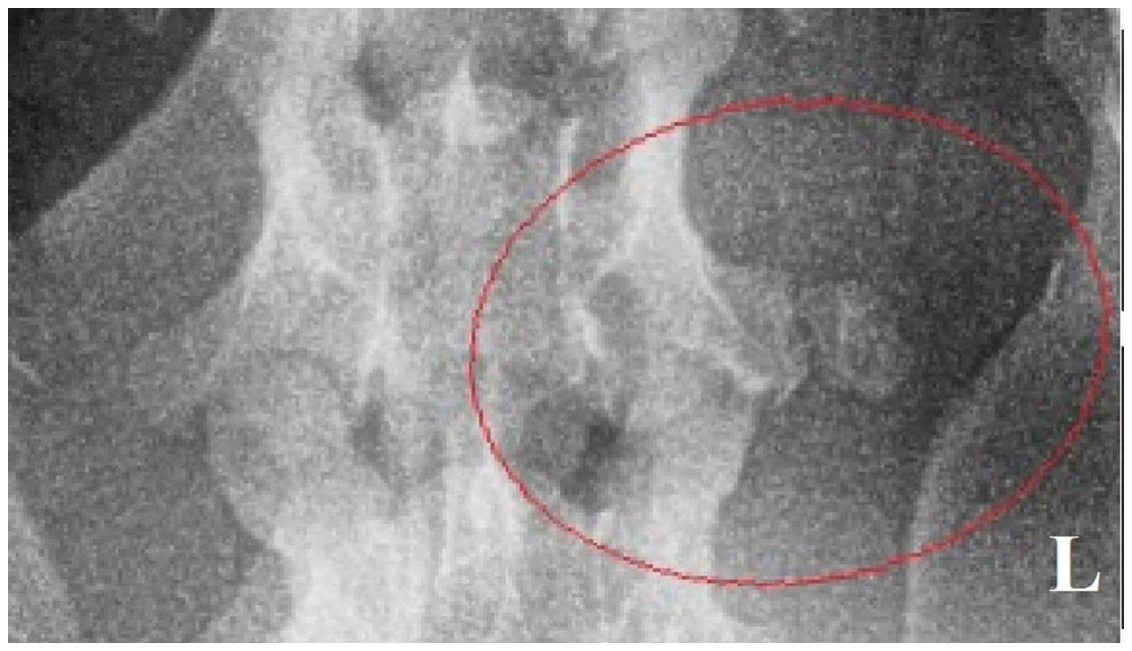
Ventrodorsal radiograph of the cervical region with a small osseous lesion after 2 years from the surgery (red ellipse).

Surgery was performed. The patient was placed in right lateral recumbency with thoracic limbs tied caudally; a skin incision was made over the mass, and the sternohyoideus and sternooccipitalis muscles were retracted. First, the firm capsule over the osseous mass was dissected. The fluid was drained, and the bony structure was exposed. Fluid and tissue samples from the lesion were submitted for aerobic and anaerobic cultures that had negative growth. The lesion was attached to the latissimus cervicis muscle and had loose, fibrous attachment to the left transverse process of the C5 vertebra. The left C6 spinal nerve was embedded in the osseous mass. The mass was dissected, freed from the fibrous attachment, and removed as one piece. The entrapped spinal nerve was meticulously and gently dissected, freed, and cleared from the osseous mass. The tissues at the site were sutured, and the wound was closed routinely. The excised tissue was submitted for histopathological examination. It measured 12 × 18 × 12 mm and appeared partially encapsulated with an irregular surface and white to light brown color. It was firm and gritty to cut (Figure 2A). Staining techniques used were hematoxylin–eosin (HE) and Giemsa. Microscopically, it presented as a well-circumscribed partially encapsulated, multiloculated formation of fully differentiated woven bone fading into more mature lamellar bone. Bone spicules were coated by a hypercellular osteoblastic lining peppered with some osteoclasts. The endosteal compartment showed central hematopoietically active red bone marrow. The capsule consisted of thick bundles of parallel-oriented, poorly cellular fibrocollagenous tissue that came into direct contact with lamellar bone. Along the capsule, there was a marked proliferation of blood vessels, both arteries and veins, with occasional cavernous spaces filled with amorphous, slightly eosinophilic material, and a variable amount of intraluminal red blood cells resembling vascular hamartoma. The blood vessel walls appeared moderately thickened, with prominent smooth muscle cells. Oligofocal metaplastic cartilage formation was observed in one section. The skeletal muscle outside the capsule appeared mild to moderately atrophic without evidence of inflammatory infiltrates. The morphological diagnosis was an intramuscular, mature HO with vascular hamartoma (Figure 3). Post-surgery radiographs detected a dense residual tissue (suspected to be the rest of the firm capsule), confirming that HO had probably not been completely removed (Figure 2B). Within 1 month post-surgery, the lameness fully resolved. Two years later, an osseous lesion of 8 mm in size was detected on routine follow-up radiographs at the surgery site; however, there were no associated clinical signs (Figure 4). Neurological examination findings at these follow-up time points were normal.

## Discussion

HO within the skeletal muscles is in human medicine typically described as fibrodysplasia ossificans progressiva (genetic form) and myositis ossificans traumatica or circumscripta (acquired form), the mechanism of which is not fully understood and is mostly suspected to be secondary to trauma. In cases of both genetic and acquired forms, a recurrence of lesion after surgical excision is common, as was observed in the described case report 2 years following the surgery. Periodical recheck radiographs were obtained because the residual tissue suggested possible regrowth and a general potential for HO recurrence. Another excision will follow if necessary (20). HO in dogs is rare, with most reported cases affecting hip joints and the appendicular muscles (9, 10, 21, 22). One similar report of HO close to the cervical spine in a dog with lameness was published, but no connection with neural structures was mentioned (8). In the case presented here, the combination of diagnostic imaging and histopathology was consistent with reports of HO in human medicine. A combination of imaging modalities (CT, MRI, and Doppler ultrasound) is used in HO diagnostic procedures in people. In our case, we used a combination of radiographs and MRI, where matured HO presents as a cancellous fat that is hyperintense on T1W and T2W images outlined by the hypointense cortical bone, which can be considered diagnostic. In this case, the HO was diagnosed at a later stage, when trabecular bone had formed. In people (compared to veterinary medicine), radiographic and histologic features of earlier HO stages (which have different characteristics) have also been described. Therefore, when MRI detects a mature HO, no further imaging is necessary. On the other hand, MRI of earlier stages of HO has a great advantage in excluding other differential diagnoses—recognizing MRI patterns in HO could be very beneficial in the early phases as the condition is commonly misdiagnosed for osteomyelitis or malignancies such as sarcomas (23–26). The histologic features mirrored radiographic findings—the early lesion shows very cellular sheets of plump fibroblasts, the cells are spindle-shaped or stellate, and they seem to float in a myxoid extracellular matrix. One week later, seams of osteoid appear in the peripheral portions. In human medicine, because of dense cellularity and osteoid production, lesions at this stage of evolution have been called “*pseudomalignant osseous tumors of soft tissue*” (27–29). Despite the high cellularity and mitotic figures, cytological atypia and abnormal mitoses are absent. A characteristic feature of HO is the entrapment of skeletal muscle in the peripheral portions of the mass. Six weeks later, the outer portion of the mass shows dense lamellar bone arranged as a pseudocortex. Six months to a year later, lesions evolve into the thick, mature trabecular bone. Bone marrow may also be present in older lesions. Complications of HO can develop as a restriction of movement of adjacent joints, and occasionally a lesion may impinge on an adjacent nerve, a pathology up to this date described only in cats (sciatic and other major nerves were encased in progressive fibrodysplasia ossificans cases) and humans (11, 30). Four factors are theorized as necessary in the pathogenesis of non-genetic HO (31). First, there must be a primary insult, usually an episode of trauma that may form a hematoma. Often, the injury is minimal and consists of only a few torn muscle or collagen fibers. The second factor is a signal from the site of injury—this signal is most probably a protein secreted from cells of the injured tissue or from inflammatory cells arriving in response to the tissue injury. Third, there must be a supply of mesenchymal cells—genes that synthesize osteoid and chondroid material are activated and cause these mesenchymal cells to differentiate into osteoblasts or chondroblasts—HO formation may occur anywhere in these soft tissues, and sites include skeletal muscles and perivascular and fibrous tissues. Finally, a necessary environment that is conducive to the continued production of heterotopic bone must be present. Of these four factors, signaling agents appear to play the most important role in the formation of heterotopic bone, and recent progress has been made in the understanding of these agents in human medicine (4, 32–37). In our case, the owner did not describe any traumatic event, but an injury during the first year of life may be speculated. Although HO may develop spontaneously, the process is initiated by trauma in 60–75% of cases in humans. HO was first described during World War I as a consequence of blast injuries and remains a major cause of morbidity in soldiers returning from conflicts in Iraq and Afghanistan (38–41). A distinctive feature is lesion maturation, which is apparent clinically, radiologically, and histologically. Our patient had episodes of swelling, pain, and signs of inflammation in the history, which is consistent with tissue irritation and HO maturation over several months. A large soft tissue swelling was evident prior to the surgery. The fluid-filled component, with variable tissue characteristics, may represent part of a hematoma, which is described as being seen in the early phases due to hemorrhage (42). HO in human medicine is staged using several classification methods, none of which are used in veterinary medicine. Nevertheless, the paravertebral area is suspicious for post-traumatic HO evolution in our case (43–46). Nerve compression or impingement has been described in stenotic disorders or traumatic events, typically spinal nerves of the lumbosacral region and obturator or sciatic nerves after pelvic fractures or surgeries (47–49). Cases of HO with nerve compression or impingement have not yet been published in dogs in any location along the vertebral column. In the case presented herein, the left C6 spinal nerve was compressed; this nerve is part of suprascapular, subscapular, and musculocutaneous nerves, and consequently, the flexor reflex of the affected thoracic limb is weakened, as was in our patient. The main limitation of the article is the lack of visual direct proof demonstrating nerve entrapment, such as an intraoperative image or sufficient MRI detail to visualize the nerve. Nevertheless, the neurological exam, MRI lesion localization, surgical findings, and resolution of the neurological deficits following surgery are all consistent with the nerve entrapment described in this case. To the authors´ knowledge, this is the first report of HO in paravertebral muscles with nerve impingement or entrapment causing neurogenic lameness in dogs (50).

## Data availability statement

The original contributions presented in the study are included in the article/supplementary material, further inquiries can be directed to the corresponding author.

## Ethics statement

Ethical review and approval was not required for this article because we are presenting a case study with routine neurological diagnostic testing (spinal MRI) and surgery approved by the owner. We did not perform any experimental treatment.

## Author contributions

IH: Writing – original draft. MR: Writing – review & editing. KM: Writing – review & editing. MB: Writing – review & editing. AC: Writing – review & editing. VP: Supervision, Writing – review & editing.
